# Microwave-Assisted Extraction of Cannabinoids in Hemp Nut Using Response Surface Methodology: Optimization and Comparative Study

**DOI:** 10.3390/molecules22111894

**Published:** 2017-11-03

**Authors:** Chih-Wei Chang, Ching-Chi Yen, Ming-Tsang Wu, Mei-Chich Hsu, Yu-Tse Wu

**Affiliations:** 1 School of Pharmacy, Kaohsiung Medical University, Kaohsiung 80708, Taiwan; wxes9050304@gmail.com (C.-W.C.); date0315@hotmail.com (C.-C.Y.); 2 Chinese Medicine Department, Ditmanson Medical Foundation, Chiayi Christian Hospital, Chiayi City 60002, Taiwan; cych07093@gmail.com; 3 Department of Sports Medicine, Kaohsiung Medical University, Kaohsiung 80708, Taiwan; meichich@kmu.edu.tw; 4 Department of Medical Research, Kaohsiung Medical University Hospital, Kaohsiung 80708, Taiwan

**Keywords:** Δ9-tetrahydrocannabinol, cannabidiol, cannabinol, marijuana, cannabis

## Abstract

Hemp nut is commonly incorporated into several food preparations; however, most countries set regulations for hemp products according to their cannabinoid content. In this study, we have developed an efficient microwave-assisted extraction (MAE) method for cannabinoids (i.e., Δ9-tetrahydrocannabinol, cannabidiol, and cannabinol) in hemp nut. Optimization of the MAE procedure was conducted through single factor experiments and response surface methodology (RSM). A comparative study was also conducted to determine the differences in the extraction yields and morphology of hemp nut between MAE and reference extraction methods, namely heat reflux extraction (HRE), Soxhlet extraction (SE), supercritical fluid extraction (SFE), and ultrasound-assisted extraction (UAE). Among the independent variables in RSM, the temperature was the most significant parameter. The optimal conditions of MAE were as follows: extraction solvent of methanol, microwave power of 375 W, temperature of 109 °C, and extraction time of 30 min. Compared with reference extraction methods, MAE achieved the highest extraction yields of total cannabinoids in hemp nut (6.09 μg/g for MAE; 4.15 μg/g for HRE; 5.81 μg/g for SE; 3.61 μg/g for SFE; 3.73 μg/g for UAE) with the least solvent consumption and shortest time. Morphological observations showed that substantial cell rupturing occurred in the microstructure of hemp nut after MAE, indicating enhanced dissolution of the target compounds during the extraction process. The MAE method is thus a rapid, economic, and environmentally friendly extraction method that is both effective and practical for industrial applications.

## 1. Introduction

Cannabis is one of the oldest domesticated crops used for medical and industrial purposes. Cannabis, which is known as marijuana for medical purposes, typically possesses a high content of cannabinoids. For industrial purposes, cannabis is known as hemp and it is cultivated to prepare foodstuffs, oils, and textiles. Currently, a continually expanding variety of hemp food products is available. Hemp seed or nut (after shell removal), which has an excellent nutritional value and pleasing nutty taste, is commonly incorporated into several food preparations, such as granola, protein powders, waffles, ice cream, tofu, snack bars, bread, yogurts, pizza, and beverages [1]. However, most countries set regulations for hemp products according to their cannabinoid content, and the legal limits vary from country to country. Although numerous cannabinoids exist in the cannabis plants, Δ9-tetrahydrocannabinol (THC), cannabidiol (CBD), and cannabinol (CBN) play a dominant role. THC is the major psychoactive cannabinoid; CBD is a non-psychoactive cannabinoid with antiepileptic properties; CBN, a degradation product of THC, is 10% as psychoactive as THC [2].

Prior to qualitative and quantitative analyses, crude plant material must undergo effective extraction. In the literature, a number of approaches have been reported for the extraction of cannabinoids from hemp products, such as Soxhlet extraction (SE) [3], homogenization [4,5], supercritical fluid extraction (SFE) [6], and ultrasound-assisted extraction (UAE) [7,8]. These methods require a long extraction time or large solvent volumes, or exhibit poor performance. Recently, microwave-assisted extraction (MAE) has been considered a sustainable “green technique”, which is a more favorable and powerful approach compared with conventional extraction techniques. Using microwaves, extraction procedures can now be completed in shorter time as well as reduced organic solvent, wastewater elimination and energy consumption [9]. With the dual mechanism of ionic conduction and dipole rotation, microwave energy can rapidly heat solvents to extract analytes from the sample matrix into the solvents [10]. Hence, MAE not only increases the efficiency but benefits environmental preservation by preventing pollution and reduces the processing cost.

MAE has been successfully applied in extracting various substances from plant matrices, including oil from green coffee beans (*Coffea arabica* L.) [11], catechins and caffeine from tea (*Camellia sinensis* L.) [12], and flavonoids from lemon (*Citrus limon* L.) [13], to pesticides, organic pollutants, phenols, metals, and polymers [14]. However, no report has described the application of MAE to cannabinoid analysis.

For an efficient MAE technique, it is necessary to optimize the conditions and design strategy. Response surface methodology (RSM) is a statistical and mathematical technique for developing, improving, and optimizing the design process [14] and has been widely utilized in the optimization of extraction processes, such as heat reflux extraction (HRE) [15], SFE [16], and UAE [17,18]. In this study, we developed a simple and reproducible coupled MAE and HPLC-EC method for analyzing cannabinoids in hemp nut. Independent factor optimization of MAE was conducted through single factor experiments and RSM. A comparative study was also conducted to determine the differences in the yield of cannabinoids and the morphology of hemp nut between MAE and reference extraction methods, namely HRE, SE, SFE, and UAE. 

## 2. Results and Discussion

### 2.1. Screening of Microwave-Assisted Extraction Parameters using Single Factor Experimental Design

A single factor experimental design was applied for selecting the levels of various influencing factors in MAE, including the extraction solvent and the extraction conditions, namely, microwave power, temperature, and extraction time.

#### 2.1.1. Effect of Extraction Solvent on the Yields of Cannabinoids

**Figure 1:**
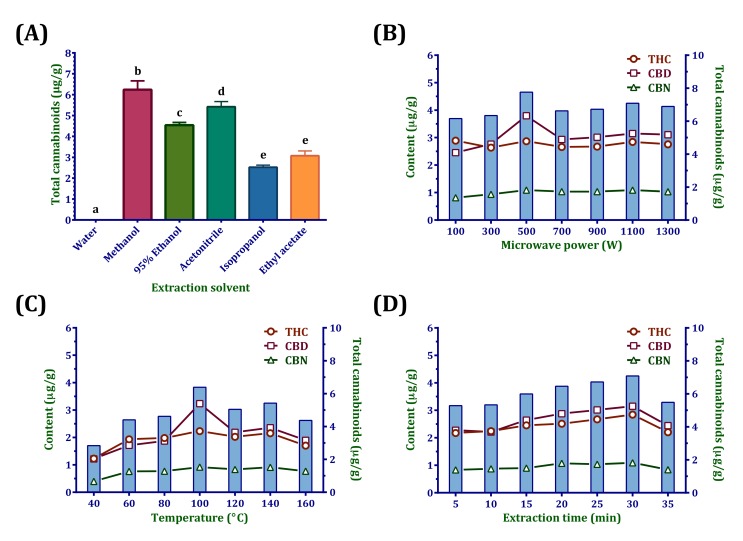
Effect of (**A**) extraction solvent, (**B**) microwave power, (**C**) temperature, and (**D**) extraction time on the yields of cannabinoids. Different letters (a, b, c, d, e) indicate a significant difference (*p* < 0.05).

The choice of an appropriate solvent is a basic factor that affects a microwave (or any other) extraction process. The selected solvent should have a strong ability to absorb microwaves, high efficiency in converting microwaves into heat, a strong ability to dissolve the analytes of interest, high interactivity with the matrix, high selectivity toward the analytes, and low selectivity toward unwanted matrix components [10,14]. In our study, we adjusted the usage of the extraction solvent while the microwave power, temperature, and extraction time were kept at 700 W, 100 °C, and 20 min, respectively. As shown in Figure 1A, water as an extraction solvent produced the lowest yield of cannabinoids, which can be attributed to their poor solubility. Furthermore, because water has the largest dielectric constant (a measure of the efficiency in converting microwave energy into heat), the sample was found to be scorched after high-temperature heating. Methanol as an extraction solvent achieved the highest yield of cannabinoids. Acetonitrile achieved the second highest, 95% ethanol achieved the third highest, and isopropanol and ethyl acetate achieved the fourth highest yields of cannabinoids. 

While some studies [19,20] have utilized MeOH/CHCl_3_ 9:1 (*v*/*v*) as extraction solvent for cannabinoids, Brighenti et al. [21] have revealed that alcohols are more suitable extraction solvents compared with the less polar solvents (i.e., acetone, MeOH/CHCl_3_ 9:1 (*v*/*v*), and hexane) in dynamic maceration. Our previous study [2] has shown that using 60% isopropanol in UAE might yield slightly more amounts in cannabinoids compared with using methanol. However, another major concern in addition to the polar nature is its, the mixed solvent, uncertainty in response to the microwave. It has been acknowledged that a small change in water percentage of mixed solvent can substantially alter the microwave absorption and electrical conductivity [22]. Taken together, we chose methanol for subsequent MAE processes.

#### 2.1.2. Effect of Microwave Power on the Yields of Cannabinoids

In this single factor experiment, we adjusted the microwave power while the temperature and extraction time were kept at 100 °C and 20 min, respectively. The microwave power can affect the time required to reach the set temperature during extraction. In general, a low or moderate power with longer exposure is selected to avoid a “bumping” phenomenon generating a high power output [10]. However, in our experiment, as shown in Figure 1B, we found that 100 °C could barely be reached at 100 W. The cannabinoid yields were mildly influenced by the microwave power. When the microwave power was 500 W, the concentration of cannabinoids was slightly higher than that at any other power. Thus, a microwave power of 300–1300 W was applied in the RSM process.

#### 2.1.3. Effect of Temperature on the Yields of Cannabinoids

In this single factor experiment, we adjusted the temperature while the microwave power and extraction time were kept at 700 W and 20 min, respectively. The temperature is the most investigated parameter in MAE. In the closed vessels, the tested temperature exceeded the boiling point of methanol, which is 65 °C, resulting in high extraction efficiency. When temperature rose, the desorption of analytes from the sample to the matrix increased; moreover, increasing the temperature improved sample wetting and matrix penetration by reducing the surface tension and solvent viscosity [10]. As shown in Figure 1C, 40 °C produced the lowest efficiency, whereas 100 °C provided the highest yield. Conversely, setting a much higher temperature degraded the cannabinoids. Thus, a temperature of 80–160 °C was applied in the RSM process.

#### 2.1.4. Effect of Extraction Time on the Yields of Cannabinoids

In this single factor experiment, we adjusted the extraction time while the microwave power and temperature were kept at 700 W and 100 °C, respectively. The extraction times of MAE are extremely short compared with those of conventional methods. A long extraction time is associated with a risk of degradation of thermolabile components. Often 3–10 min is sufficient; however, in our case, the cannabinoid content increased when the extraction time was increased from 10 to 30 min (Figure 1D). By contrast, the yield decreased at 35 min. Thus, an extraction time of 10–30 min was applied in the RSM process.

#### 2.1.5. The Relationship between the Studied and the Other Cannabinoids in Microwave-Assisted Extraction

Apart from the three studied cannabinoids (i.e., THC, CBD, and CBN), non-psychoactive carboxylated forms, Δ9-tetrahydrocannabinolic acid A (THCA-A) and cannabidiolic acid (CBDA), are present in fresh herbal material as well. A previous study has shown that increased extraction yields of cannabinoids could be observed in the boiling water. The yields reaches the peak at 15 min and declines after 30 min, which the finding is in agreement with our study. Although an adequate amount of heat produces a higher yield, it is plausible that heat can also cause degradation by breaking the cannabis terpenes [23]. On the other hand, when exposed to a high temperature (145 °C for 30 min), THCA-A and CBDA progressively decarboxylate to THC and CBD, respectively, and THC is further converted to CBN to a small degree. These changes therefore lead to an increase in the extraction yields of THC, CBD, and CBN. In some circumstances, CBDA can be completely transformed into CBD [23]. In the present study, although the abovementioned changes should be taken into account, we might not be able to precisely calculate all portions of the chemical transformations during heating.

### 2.2. Optimization of Microwave-Assisted Extraction Conditions using Response Surface Methodology

The input factors and their levels for RSM were selected on the basis of the single factor experiments, a Box–Behnken design (BBD) with three variables and three levels was adopted.

#### 2.2.1. Statistical Analysis and Model Fitting

Seventeen experimental runs were performed to investigate the reactive extraction process. The experimental design, variables, coded and decoded levels, and responses are listed in Table 1. After fitting the results into the second-order polynomial model (1), the Equations (2)–(4) for extraction yields of analytes in coded factors are generated as follows:

(1) $$Y=\;\beta_{0}+\;\overset{3}{\underset{i=1}{\sum }}\beta_{i}X_{i}+\;\overset{3}{\underset{i=1}{\sum }}\beta_{ii}X_{i}^{2}+\;\overset{2}{\underset{i=1}{\sum }}\overset{3}{\underset{j=i+1}{\sum }}\beta_{ij}X_{i}X_{j}$$

(2) $$Y_{\mathrm{THC}}=2.2-0.012X_{1}-0.37X_{2}+0.083X_{3}-0.32X_{2}^{2}$$

(3) $$Y_{\mathrm{CBD}}=2.41-0.049X_{1}-0.1X_{2}+0.057X_{3}-0.52X_{2}^{2}$$

(4) $$Y_{\mathrm{CBN}}=0.97+0.002887X_{1}-0.071X_{2}-0.00449X_{3}-0.18X_{2}^{2}$$

where *Y* is the response value (the extraction yields of THC, CBD, and CBN) ; *β*_0_, *β_i_*, *β_ii_*, and *β_ij_* are the regression coefficients estimated using the equation for the intercept, linearity, square, and interaction, respectively; and *X_i_* and *X_j_* represent the independent variables (*X*_1_ = microwave power, *X*_2_ = temperature, *X*_3_ = extraction time).

The results of analysis of variance (ANOVA) for analyzing the experimental data and examining the statistical significance of the model terms are listed in Table 2.

High model *F* values (of THC, CBD, and CBN) and low values of *p* (<0.05) for all analytes indicated that the developed models were significant and the variations in the response can be explained by the regression equation. A nonsignificant *F* value for the lack of fit of THC, CBD, and CBN suggested that it is not related to pure error. The *p* values for the lack of fit were not significant for all three analytes (>0.05), which indicated that the models were reliable and adequate for use in predicting the responses.

#### 2.2.2. Effect of Process Factors on the Yields of Cannabinoids

**Figure 2:**
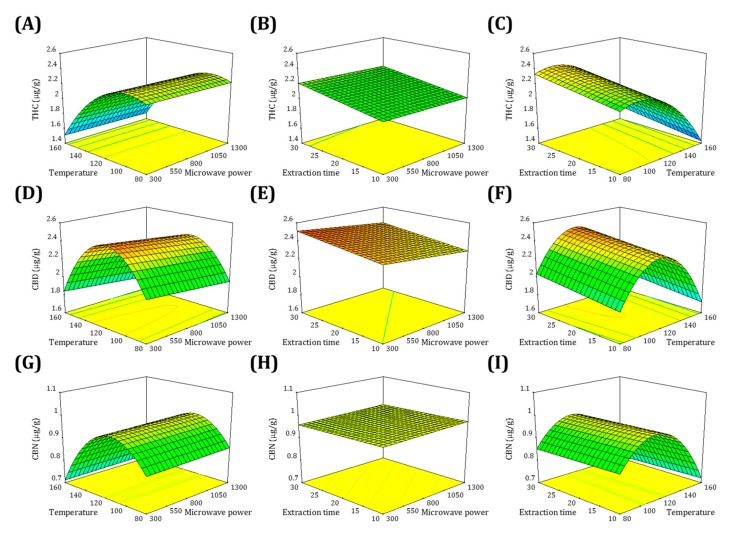
Response surface plots showing the interaction effects of microwave power, temperature, and extraction time on the yields of THC (**A**–**C**), CBD (**D**–**F**), and CBN (**G**–**I**).

**Figure 3:**
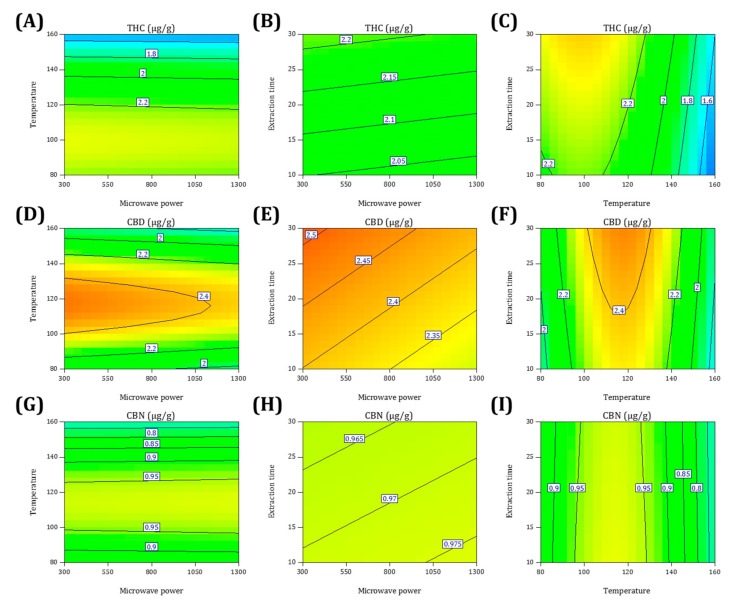
Contour plots showing the interaction effects of microwave power, temperature, and extraction time on the yields of THC (**A**–**C**), CBD (**D**–**F**), and CBN (**G**–**I**).

Figure 2 and Figure 3 show the three-dimensional response surface plots and contour plots, which illustrate the main interactive effects produced for each pair of factors. Each figure depicts the influence of two factors on the extraction yields while the third factor was fixed at the zero level, which was 800 W for microwave power (*X*_1_), 120 °C for temperature (*X*_2_), and 20 min for extraction time (*X*_3_).

Regarding the THC extraction yield, the effects of *X*_1_ and *X*_2_ are illustrated in Figure 2A and Figure 3A. *X*_2_ has a more crucial influence than that of *X*_1_. A higher yield was observed at a lower *X*_2_; however, *X*_1_ showed no significant influence on the yield. When *X*_2_ exceeded 120 °C, the yield dramatically decreased. Figure 2B and Figure 3B show the effects of *X*_1_ and *X*_3_. When *X*_1_ was kept at low or medium levels, the extraction yield was enhanced following the increase of *X*_3_, and reached a maximum. Figure 2C and Figure 3C display the interaction between *X*_2_ and *X*_3_. A higher yield was achieved at an *X*_2_ of 90–110 °C with an *X*_3_ of 30 min. We conclude that the maximal THC yield can be obtained at a microwave power of 300 W, a temperature of 96 °C, and an extraction time of 30 min.

Regarding the CBD extraction yield, the effects of *X*_1_ and *X*_2_ are illustrated in Figure 2D and Figure 3D. A half-elliptical contour plot indicated that the peak yield was located on the margin. A lower level of *X*_1_ may produce a higher yield. With an increase of *X*_2_, the extraction yield gradually increased, reaching the peak value at approximately the medium point of *X*_2_, and then declined. Figure 2E and Figure 3E display the interaction between *X*_1_ and *X*_3_. The yield was enhanced with a decrease of *X*_1_ and an increase of *X*_3_; therefore, the peak yield existed on the corner. Figure 2F and Figure 3F show the effects of *X*_2_ and *X*_3_. A half-elliptical shape again appeared on the contour plot. Such findings indicate that when *X*_2_ was kept within 100–140 °C, the yield increased as *X*_3_ increased. The maximal yield appeared on the margin of the highest *X*_3_ and at approximately the medium point of *X*_2_. We conclude that the maximal CBD yield can be obtained at a microwave power of 300 W, a temperature of 116 °C, and an extraction time of 30 min.

Regarding the CBN extraction yield, the effects of *X*_1_ and *X*_2_ are illustrated in Figure 2G and Figure 3G. The CBN response was similar to the THC response. *X*_2_ exhibited a substantial effect on the yield, whereas *X*_1_ showed relatively little influence; however, the maximal yield existed at approximately the medium point of *X*_2_. Figure 2H and Figure 3H show the interaction between *X*_1_ and *X*_3_, which exhibited a typically opposite effect on the CBD response. The continual rise of the yield corresponding to an increase of *X*_1_ can be attributed to the fact that the lower *X*_1_ was not sufficient for CBD extraction. When the yield was enhanced with a higher *X*_1_ and a lower *X*_3_, the peak yield was located on the opposite corner to that of CBD. Figure 2I and Figure 3I display the effects of *X*_2_ and *X*_3_. Within 100–130 °C, a lower *X*_2_ was associated with a higher yield. We conclude that the maximal CBN yield can be obtained at a microwave power of 1300 W, a temperature of 112 °C, and an extraction time of 10 min.

#### 2.2.3. Confirmation of Optimal Microwave-Assisted Extraction Conditions

The selected factors exhibited different influences on the yields of the three cannabinoids; however, when all cannabinoids were considered simultaneously, the predicted optimal conditions for cannabinoid extraction were as follows: extraction solvent of methanol, microwave power of 375 W, temperature of 109 °C, and extraction time of 30 min. After three parallel experiments were performed, the residuals (%) between the predicted and observed responses were calculated as (observed value − expected value)/expected value × 100%. The responses were in close agreement (2.8–9.3% of residual) under the optimal conditions, as shown in Table 3, indicating the reliability of the established models.

### 2.3. Comparison of Microwave-Assisted Extraction with Reference Extraction Methods

**Figure 4:**
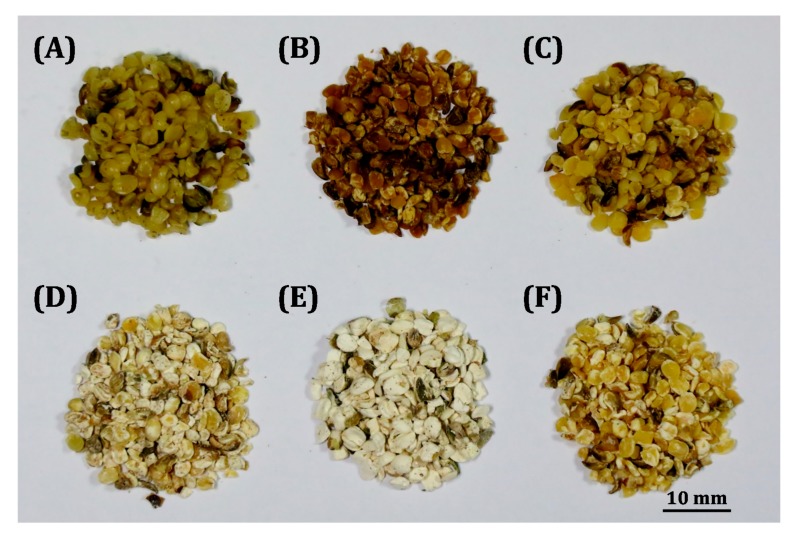
Images of (**A**) crude hemp nut and hemp nut after (**B**) MAE, (**C**) HRE, (**D**) SE, (**E**) SFE, and (**F**) UAE.

To validate the proposed MAE method, its efficiency in the extracting cannabinoids from hemp nut was compared with those of HRE, SE, SFE, and UAE. Images of crude and treated hemp nut are shown in Figure 4.

**Figure 5:**
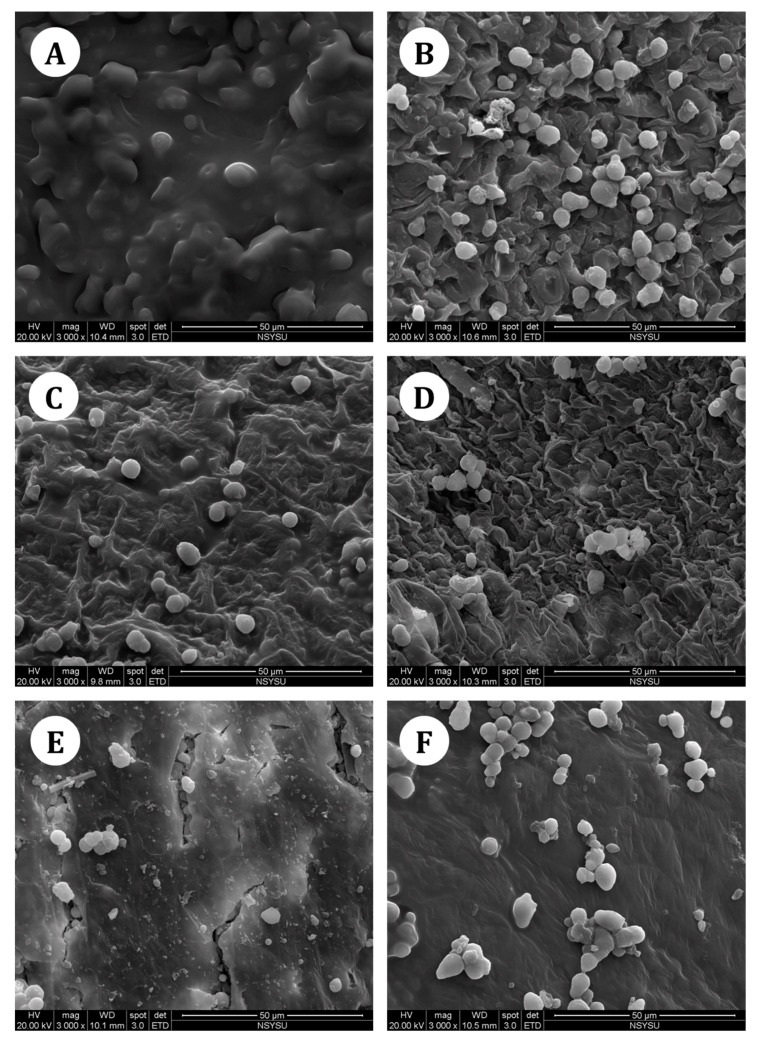
Scanning electron microscopy images of (**A**) crude hemp nut and hemp nut after (**B**) MAE, (**C**) HRE, (**D**) SE, (**E**) SFE, and (**F**) UAE.

Crude hemp nut is plump and shiny, indicating high contents of protein, oil, and other constituents (Figure 4A). The presence of numerous oil cells with a complete balloon shape was confirmed using the SEM (Figure 5A); however, they were completely disrupted after MAE.

Microwaves can penetrate the plant matrix and generate heat within the cell to facilitate cell rupture, thus enhancing the dissolution of target compounds in the extraction solvent within a short time [24]. After the MAE treatment, the cell walls of the hemp nut were completely broken, resulting in substantial cell shapes, because of the sudden temperature rise (Figure 5B). HRE was used as a distillation technique for transferring the solvent to the sample, and the compounds were extracted through the permeation and solubilization processes [25]. The sample underwent minor destruction after HRE treatment (Figure 5C). For SE, the sample was placed in a fiber thimble and repeatedly percolated with condensed vapors of the solvent. In each cycle, the sample was extracted with a pure solvent, which has a strong capability to dissolve the target compounds. Long-duration repeated extraction wrinkled the cells of hemp nut considerably (Figure 5D). Regarding SFE, the supercritical fluids dissolved the compounds through diffusion. Diffusion is considerably faster in supercritical fluids than in liquids, because there is no surface tension and the viscosity is much lower than that of liquids [26]. SFE can extract oily constituents under high pressure, and therefore, some marked cracks were found in the microstructure of the sample (Figure 5E). For UAE, ultrasound supplied high frequency oscillation to the solvent, and the solvent flowed rapidly to generate constant pressure [27]. The superficial constituents of the samples were dissolved into the solvent; however, no remarkable disruption of cells was observed (Figure 5F).

In these extraction techniques, MAE extracted the maximal amounts of CBD and CBN (*p* < 0.05 compared with the others), whereas SE extracted the maximal amount of THC (*p* < 0.05 compared with the others) (Table 4). Overall, MAE achieved the highest extraction yields for total cannabinoids in hemp nut. It exhibited a shorter extraction time, required a lesser amount of solvent, and entailed a simpler operation process. Moreover, it enabled up to 40 samples to be processed in a single run.

### 2.4. Possible Industrial Applications for the Developed Microwave-Assisted Extraction

Using microwaves for extracting bioactive compounds from herbal materials has several important merits [28]. On the one hand, MAE adopts electrical power instead of fossil fuel allowing a sustainable source and a safe working condition. On the other hand, microwave is a non-contact heat source, which minimizes thermal gradient and decreases equipment size. While the energy transfer is accelerated, the heating becomes more effective.

Since there are numerous commercial cannabis-derived products, governments have set laws regulating the threshold of cannabinoids content in order to prevent their abuse. For this legal aspect, a rapid, effective and environmentally friendly extraction method coupled with a sensitive quantitative method may be attractive. However, apart from using MAE as one of the techniques for quality control, there are also high demands for cannabinoids for medical purposes. For instance, THC and CBD are particular useful in relieving neuropathic pain [29], anorexia [30], and epilepsy [31]. Our efficient MAE method could be used in a process to treat a complex biomass to obtain valuable compounds. Nonetheless, a specific restriction of industrial MAE methods is that the structure of raw material is a key factor influencing the extractability. Further study regarding the application of MAE method in different matrices is warranted.

## 3. Material and Methods

### 3.1. Chemical and Reagents

Chemical standards including THC, CBD, and CBN were purchased from Sigma–Aldrich (St. Louis, MO, USA). Dodecyl 4-hydroxybenzoate was purchased from Tokyo Chemical Industry (Tokyo, Japan). Pure water was prepared through a Milli-Q system (Millipore, Bedford, MA, USA). Methanol was obtained from Duksan (Gyeonggi-do, Korea). Isopropanol and 95% ethanol were obtained from Echo Chemical (Miaoli, Taiwan). Acetonitrile was obtained from Avantor (Center Valley, PA, USA). Ethyl acetate was obtained from Honeywell (Morris Plains, NJ, USA). A phosphate buffer was obtained from Merck (Darmstadt, Germany).

### 3.2. Instrumentation

A voltammetric behavior experiment was performed using a 621E electrochemical analyzer (CH Instruments, Austin, TX, USA). The identification and quantification of cannabinoids were conducted using a high performance liquid chromatography and electrochemical detection (HPLC-EC) system consisting of a 5160 pump and a 5260 auto sampler (Hitachi, Tokyo, Japan) coupled with a LC-4C amperometric detector (BAS, West Lafayette, IN, USA). MAE experiments were performed using a MARS 5 microwave system (CEM, Matthews, NC, USA). UAE experiments were performed using a 3210 ultrasonic system (Branson, Danbury, CT, USA). SFE experiments were performed using a *Spe-ed* SFE supercritical fluid system consisting of an SFE oven module, SFE pump module, and SFE control and collection module (Applied Separations, Allentown, PA, USA) coupled with air compressors (JW, Taichung, Taiwan). A D-606 cooling bath (Deng Yng, New Taipei, Taiwan) was applied for HRE, SE, and SFE experiments. Microstructure analysis was performed using a Quanta 200 environmental scanning electron microscope (SEM, FEI, Hillsboro, OR, USA). A 108 Sputter Coater (Cressington, Watford, Hertfordshire, UK) was used for gold coating. 

### 3.3. Analytical Methods

#### 3.3.1. Voltammetric Behavior Experiment

**Figure 6:**
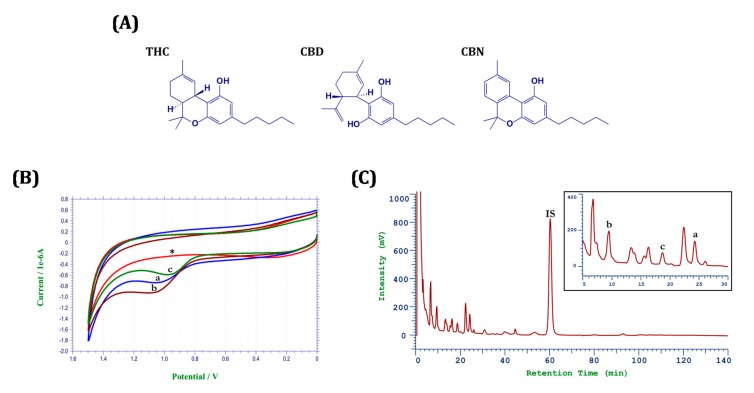
(**A**) Chemical structures, (**B**) electrochemical characterization, and (**C**) HPLC-EC chromatogram for cannabinoids. *: blank, a: THC, b: CBD, c: CBN, IS: internal standard.

The voltammetric behavior experiment was conducted to obtain the peak potential for THC, CBD, and CBN (Figure 6A). A glassy carbon working electrode, an Ag/AgCl reference electrode, and a Pt counter electrode were used. The electrode was first activated in 15 mL of 0.1 M phosphate buffer (pH 2.5) through cyclic voltammetry (CV) sweeps until a stable response was obtained. An amount of 10 μg of each analyte was separately added to the buffer. After stirring for 3 min, a potential scan was initiated and the CV response was recorded between 0 and +1.5 mV with a scan rate of 100 mV/s. The CV results from the voltammetric behavior experiment are summarized in Figure 6B. When the analytes were separately added to the electrolyte, an anodic peaks for THC, CBD, and CBN were observed at peak potentials of +1.082 V, +1.093 V, and +0.976 V, respectively. Therefore, the working potential for further HPLC-EC analysis was set at +1.1 V.

#### 3.3.2. High Performance Liquid Chromatography with Electrochemical Detection Condition

The HPLC-EC method was developed according previous study [3] with slight modification. Cannabinoid separation was conducted on a Kinetex C18 LC column (100 × 2.1 mm, i.d.; 2.6 μm, Phenomenex, Torrance, CA, USA) with a C18 guard cartridge (20 × 2.1 mm, i.d.; sub-2 μm, Phenomenex). Dodecyl 4-hydroxybenzoate served as an internal standard (IS). The isocratic mobile phase consisted of 10 mM phosphate buffer (pH 2.5)-methanol-acetonitrile (1:1:1, *v*/*v*/*v*) that was delivered at a flow rate of 0.35 mL/min. The injection volume was 20 μL. A glassy carbon working electrode and an Ag/AgCl reference electrode were used for the electrochemical detector. The potential was set at +1.1 V, the filter was set at 0.1 Hz, and the range was set at 20 nA. The concentration of each analyte was corrected and calculated according to the ratio of the peak area of each analyte to that of the corresponding IS. 

The HPLC-EC chromatogram for the extracts from hemp nut after MAE is shown in Figure 6C. The retention time for THC, CBD, CBN, and IS was 24.3 min, 9.5 min, 20.2 min, and 60.3 min, respectively. The analytical method was validated for linearity, intra- and interday variations, and recovery. As presented in Table S1, high linearity (*r*^2^ > 0.9998) was observed in the range of 3.91–500 ng/mL. The accuracy ranged from 93.1% to 108.3%; the precision, as indicated by the relative standard deviations, ranged from 1.5% to 7.4% (Table S2). The recoveries for THC, CBD, CBN were 76.8 ± 7.9%, 87.7 ± 7.9%, and 60.9 ± 5.0%, respectively (Table S2).

### 3.4. Extraction Methods

#### 3.4.1. Microwave-Assisted Extraction 

Crude hemp nut was purchased from Bama, Guangxi, China (September 2015). The sample (1.0 g) was mixed with 12 mL of methanol in a closed vessel and irradiated with microwave power (375 W) at 109 °C for 30 min. After cooling, the extract was filtered through filter paper (Advantec, Tokyo, Japan) into a 20-mL volumetric flask. The residue was washed with methanol and followed by addition to volume. The final extract was then filtered through a syringe filter (PTFE membrane, 0.2 μm, Phenomenex) and stored at −20 °C for HPLC-EC analysis.

#### 3.4.2. Heat Reflux Extraction 

The sample (5.0 g) was mixed with methanol (100 mL) in a round-bottom flask and boiled at 90 °C for 4 h. The temperature of the cooling bath used in the condenser was set at 15 °C. After cooling, the extract was filtered and quantitatively adjusted to 100 mL. The subsequent processes were the same as those described previously.

#### 3.4.3. Soxhlet Extraction 

The sample (15.0 g) was placed in a paper thimble (Advantec) and placed in a Soxhlet apparatus. In a round-bottom flask, 300 mL of methanol was added and used for 8-h extraction at 90 °C. The temperature of the cooling bath used in the condenser was set at 15 °C. After cooling, the extract was filtered and quantitatively adjusted to 300 mL. The subsequent processes were the same as those described previously.

#### 3.4.4. Supercritical Fluid Extraction 

The sample (2.5 g) was first transferred to a metal extractor vessel housed in an oven with the temperature set at 50 °C. Supercritical carbon dioxide was used as a solvent for extraction under 5000 psi. The output valve temperature was maintained at 120 °C. The temperature of the cooling bath was set at 4 °C. After 30-min static extraction, 90-min dynamic extraction was conducted with an air flow rate of 2.5 L/min. Afterwards, the extract was collected in a glass vial containing methanol and then filtered and quantitatively adjusted to 50 mL. The subsequent processes were the same as those described previously.

#### 3.4.5. Ultrasound-Assisted Extraction 

The sample (1.0 g) was mixed with 20 mL of methanol in a tube and ultrasonicated for 30 min at 47 kHz. The extract was filtered and quantitatively adjusted to 20 mL. The subsequent processes were the same as those described previously.

### 3.5. Experimental Design

#### 3.5.1. Single Factor Experimental Design

First, the extraction solvent (water, methanol, 95% ethanol, acetonitrile, isopropanol, and ethyl acetate) was studied. The optimal solvent was chosen for further investigation, and the extraction conditions, namely, a microwave power of 100–1300 W, temperature of 40–160 °C, and extraction time of 5–35 min, were determined. The factors were fixed at their median values (700 W, 100 °C, and 20 min) when the other factors were investigated.

#### 3.5.2. Optimization through Response Surface Methodology 

A BBD with three variables and three levels was adopted for RSM. According to the results of the single factor experiment, three independent variables, microwave power (*X*_1_), temperature (*X*_2_), and extraction time (*X*_3_) with three levels for each, coded −1, 0, and +1 for low, medium, and high, respectively. As shown in Table 1, a total of 17 experimental runs consisting of five central points were generated. The THC, CBD, and CBN contents were chosen as the response values, and the results were fit to a second-order polynomial model (Equation (1)), where *Y* is the response value (the extraction yields of THC, CBD, and CBN); *β*_0_, *β_i_*, *β_ii_*, and *β_ij_* are the regression coefficients estimated using the equation for the intercept, linearity, square, and interaction, respectively; and *X_i_* and *X_j_* represent the independent variables. Three-dimensional response surface plots and contour plots were constructed for interpreting interactions among all independent variables and their corresponding effects. To produce the optimal magnitude of the extraction yield a “biggest-is-best” criterion was applied for each response.

Finally, in the confirmation study, three parallel experiments were conducted by using the optimal extraction conditions with the highest desirability for practically verifying the improvement of the quality characteristic.

### 3.6. Scanning Electronic Microscopy

The SEM was used to examine the morphologies of the samples after extraction through different methods. After removing the extracted solvent, the sample surfaces were first fixed on an adhesive tape and then sputter coated with a thin layer of gold. All samples were observed using the SEM under a vacuum condition at an accelerating voltage of 20.0 kV and magnification of 3000×.

### 3.7. Statistical Analysis

The data are expressed as the mean ± standard deviation. ANOVA for RSM was performed using the Design-Expert 6.0.3 software (StatEase Inc., Minneapolis, MN, USA). The effects of different solvents and extraction methods on the yield of cannabinoids were compared statistically by using ANOVA with Tukey’s *post hoc* test through the SPSS 19.0 software (International Business Machines Corporation, Armonk, NY, USA). A value of *p* < 0.05 was considered to indicate statistical significance.

## 4. Conclusions

In this study, we first developed an MAE method followed by a validated HPLC-EC technique for analyzing cannabinoids in hemp nut. Single factor experiments combined with RSM were employed for MAE process optimization. Among the independent variables in RSM, the temperature was the most significant parameter. The optimal conditions of MAE were as follows: extraction solvent of methanol, microwave power of 375 W, temperature of 109 °C, and extraction time of 30 min. Compared with the reference extraction methods, namely HRE, SE, SFE, and UAE, the proposed MAE method achieved the highest extraction yield of cannabinoids in hemp nut with the least solvent consumption and shortest time. Substantial cell ruptures were also found in the microstructure of hemp nut after MAE in morphological observation. The MAE method is thus a rapid, economic, and environmentally friendly extraction method that is effective and practical for industrial applications.

## Figures and Tables

**Table 1:** Experimental parameters of Box–Behnken design and extraction yields of three cannabinoids.

Run	Factor (Coded)			Measured Response		
	*X*_1_ (W)	*X*_2_ (°C)	*X*_3_ (min)	THC (μg/g)	CBD (μg/g)	CBN (μg/g)
1	300 (−1)	80 (−1)	20 (0)	2.18	1.79	0.79
2	1300 (+1)	80 (−1)	20 (0)	2.13	1.78	0.78
3	300 (−1)	160 (+1)	20 (0)	1.77	2.14	0.82
4	1300 (+1)	160 (+1)	20 (0)	1.44	1.65	0.85
5	300 (−1)	120 (0)	10 (−1)	2.13	2.19	0.89
6	1300 (+1)	120 (0)	10 (−1)	2.38	2.57	0.98
7	300 (−1)	120 (0)	30 (+1)	2.19	2.55	1.05
8	1300 (+1)	120 (0)	30 (+1)	2.23	2.28	0.96
9	800 (0)	80 (−1)	10 (−1)	2.05	2.16	0.98
10	800 (0)	160 (+1)	10 (−1)	1.35	1.51	0.65
11	800 (0)	80 (−1)	30 (+1)	2.65	2.22	0.90
12	800 (0)	160 (+1)	30 (+1)	1.51	1.83	0.55
13	800 (0)	120 (0)	20 (0)	2.40	2.17	0.90
14	800 (0)	120 (0)	20 (0)	2.21	2.57	1.14
15	800 (0)	120 (0)	20 (0)	1.94	2.40	0.89
16	800 (0)	120 (0)	20 (0)	2.21	2.33	0.95
17	800 (0)	120 (0)	20 (0)	2.15	2.61	0.96

*X*_1_: microwave power, *X*_2_: temperature, *X*_3_: extraction time. THC; Δ9-tetrahydrocannabinol, CBD; cannabidiol, CBN; cannabinol.

**Table 2:** Analysis of variance of reduced quadratic model for extraction yields of three cannabinoids.

Source	Sum of Squares	df	Mean Square	*F*-Value	*p*-Value	Remarks
**THC**						
Model	1.57	4	0.39	11.48	0.0005	Significant
Residual	0.41	12	0.034			
Lack of fit	0.30	8	0.038	1.41	0.3922	Not significant
Pure error	0.11	4	0.027			
Cor total	1.98	16				
**CBD**						
Model	1.28	4	0.32	6.68	0.0046	Significant
Residual	0.58	12	0.048			
Lack of fit	0.45	8	0.056	1.68	0.3104	Not significant
Pure error	0.13	4	0.032			
Cor total	1.86	16				
**CBN**						
Model	0.18	4	0.045	3.84	0.0310	Significant
Residual	0.14	12	0.012			
Lack of fit	0.10	8	0.013	1.27	0.4350	Not significant
Pure error	0.039	4	0.009851			
Cor total	0.32	16				

THC: Δ9-tetrahydrocannabinol, CBD: cannabidiol, CBN: cannabinol, df: degree of freedom.

**Table 3:** Observed and predicted levels for optimal extraction conditions.

Factor	Optimal Level		
*X*_1_ (W)	375		
*X*_2_ (°C)	109		
*X*_3_ (min)	30		
**Response**	**Predicted (μg/g)**	**Experimental (μg/g, *n* = 3)**	**Residual (%)**
THC (μg/g)	2.38	2.47 ± 0.28	3.8%
CBD (μg/g)	2.49	2.56 ± 0.17	2.8%
CBN (μg/g)	0.97	1.06 ± 0.16	9.3%

Residual (%) = (observed value − expected value)/expected value × 100%. *X*_1_: microwave power, *X*_2_: temperature, *X*_3_: extraction time. THC: Δ9-tetrahydrocannabinol, CBD: cannabidiol, CBN: cannabinol.

**Table 4:** Comparison of extraction yields for different extraction methods.

Extraction Method	Requirement			Yield (μg/g, *n* = 3)			
	Sample (g)	Solvent Consumption	Extraction Time (min)	THC	CBD	CBN	Total
MAE	1.0	12 mL of methanol	30	2.47 ± 0.28 ^a^	2.56 ± 0.17 ^a^	1.06 ± 0.16 ^a^	6.09 ± 0.47 ^a^
HRE	5.0	100 mL of methanol	240	2.23 ± 0.18 ^a^	1.32 ± 0.11 ^b^	0.61 ± 0.04 ^b^	4.15 ± 0.33 ^b^
SE	15.0	300 mL of methanol	480	3.19 ± 0.09 ^b^	1.90 ± 0.08 ^c^	0.73 ± 0.02 ^b^	5.81 ± 0.17 ^a^
SFE	2.5	225 L of carbon dioxide	120	1.97 ± 0.06 ^a^	1.08 ± 0.11 ^b^	0.57 ± 0.04 ^b^	3.61 ± 0.14 ^b^
UAE	1.0	20 mL of methanol	30	2.08 ± 0.25 ^a^	1.09 ± 0.04 ^b^	0.70 ± 0.20 ^b^	3.73 ± 0.31 ^b^

Different letters (a, b, c) indicate a significant difference (*p* < 0.05) between extraction methods. THC: Δ9-tetrahydrocannabinol, CBD: cannabidiol, CBN: cannabinol, MAE: microwave-assisted extraction, HRE: heat reflux extraction, SE: Soxhlet extraction, SFE: supercritical fluid extraction, UAE: ultrasound-assisted extraction.

## Supplementary Materials

The supplementary materials are available online.
