# Supplementary material for: Microwave-Assisted Extraction of Cannabinoids in Hemp Nut Using Response Surface Methodology: Optimization and Comparative Study
Source: Molecules. 2017 Nov 3;22(11):1894. doi: 10.3390/molecules22111894 (PMC6150297; doi:10.3390/molecules22111894)
Supplement: Supplementary file 1 [file molecules-22-01894-s001.pdf]

Table S1. Regression equation, coefficients (r^2), and linearity ranges of analytes

Analyte	Regression equation	r^2	Linear range (ng/mL)
THC	$y = 0.0006x + 0.0002$	0.9998	1.95–500
CBD	$y = 0.0006x + 0.0005$	0.9998	3.91–500
CBN	$y = 0.0008x + 0.00006$	0.9999	3.91–500

THC; Δ9-tetrahydrocannabinol, CBD; cannabidiol, CBN; cannabinol, RSD

Table S2. Intra- and interday variations and recoveries of analytes

Analyte	Intra-day (n = 6)		Inter-day (n = 6)		Recovery (n = 3)
	Accuracy (%)	Precision (RSD, %)	Accuracy (%)	Precision (RSD, %)	(%)
THC	94.0	3.1	93.1	4.4	76.8 ± 7.9
CBD	104.6	5.0	106.7	7.3	87.7 ± 7.9
CBN	106.8	1.5	108.3	7.4	60.9 ± 5.0

THC; Δ9-tetrahydrocannabinol, CBD; cannabidiol, CBN; cannabinol, RSD; relative standard deviation.
